# Bevacizumab is associated with delayed anastomotic leak after low anterior resection with preoperative radiotherapy for rectal cancer: a case report

**DOI:** 10.1186/s40792-019-0573-1

**Published:** 2019-01-31

**Authors:** Erika Machida, Yasuyuki Miyakura, Jun Takahashi, Sawako Tamaki, Hideki Ishikawa, Fumi Hasegawa, Rina Kikugawa, Shingo Tsujinaka, Alan Kawarai Lefor, Toshiki Rikiyama

**Affiliations:** 1 Department of Surgery, Saitama Medical Center, Jichi Medical University, 1-847 Amanuma-cho, Omiya-ku, Saitamashi, Saitamaken 330-8503 Japan; 2 Department of Surgery, Jichi Medical University, Tochigi, Japan

**Keywords:** Rectal cancer, Bevacizumab, Chemo-radiotherapy, Delayed anastomotic leak

## Abstract

**Background:**

Bevacizumab is an anti-angiogenesis agent used to treat patients with metastatic colorectal cancer and is associated with a variety of complications. We present a patient with rectal cancer who developed a delayed anastomotic leak more than 5 years after undergoing low anterior resection.

**Case report:**

A 78-year-old man with hematochezia was diagnosed with two synchronous rectal cancers 7 years prior to presentation. Preoperative chemo-radiotherapy was given followed by a very low anterior resection. During follow-up, multiple lymph node metastases developed, which were treated with chemotherapy. First-line chemotherapy, capecitabine, oxizaliplatin, and bevacizumab, was given over 3 years, and second-line chemotherapy, capecitabine, irinotecan, and bevacizumab, was administered over a 3-month period. After the last treatment, the patient presented with pneumaturia and fecaluria. Computed tomography scan revealed extraluminal air between the prostate and rectum, adjacent to the anastomotic site. Ulceration and fistula formation were observed on colonoscopy, and contrast radiography demonstrated a fistula at the anastomotic site. An anastomotic-urethral fistula was diagnosed and transverse colostomy was performed.

**Conclusions:**

This patient highlights a rare late adverse event at the anastomotic site associated with bevacizumab treatment and preoperative chemo-radiotherapy. Signs and symptoms suggesting anastomotic complications should be thoroughly evaluated during bevacizumab treatment, even long after surgical resection.

## Background

Bevacizumab, a humanized monoclonal antibody that targets vascular endothelial growth factor (VEGF)-A, is an anti-angiogenesis agent used to treat patients with metastatic colorectal cancer (CRC) in combination with 5-fluorouracil (5-FU)-based chemotherapy as both first- and second-line treatment [1, 2]. Bevacizumab can lead to a variety of adverse events such as arterial thrombosis [3], hemorrhage [4], and gastrointestinal perforation [5]. In addition, bevacizumab treatment increases the risk of developing an anastomotic leak in patients with rectal cancer who underwent low anterior resection. Delayed anastomotic leak, especially more than 3 months after surgery, is rare. A small number of case reports have described this complication [6–11]. We present a patient with rectal cancer who developed a delayed anastomotic leak more than 5 years after undergoing low anterior resection who had received preoperative chemo-radiotherapy (CRT).

## Case presentation

A 78-year-old man with hematochezia was diagnosed with two synchronous rectal cancers 7 years prior to presentation. One tumor was located at the rectosigmoid junction (stage T3N1M0, well-differentiated tubular adenocarcinoma), and the second was in the distal rectum, (stage T3N1M0, well-differentiated tubular adenocarcinoma). The patient had a 10-year history of diabetes mellitus and hypertension treated with medication. No family history of CRC was noted. Physical examination was unremarkable. Preoperative CRT followed by a very low anterior resection with diverting ileostomy was performed. Preoperative CRT included 5 days of 5-FU/leucovorin infusion followed by radiation therapy delivered using the four-field technique with photon radiation administered five times per week with a daily fraction of 1.8 Gy, for a total of 40 Gy. The final pathological diagnosis revealed that the rectosigmoid cancer was ypT3N1M0, and the lower rectal cancer was ypT0N0M0 (no residual cancer, pathological complete response). The postoperative course was uneventful and the ileostomy was reversed 8 months later, after completion of postoperative adjuvant chemotherapy, which included 6 months of oral 5-FU/leucovorin.

**Fig. 1:**
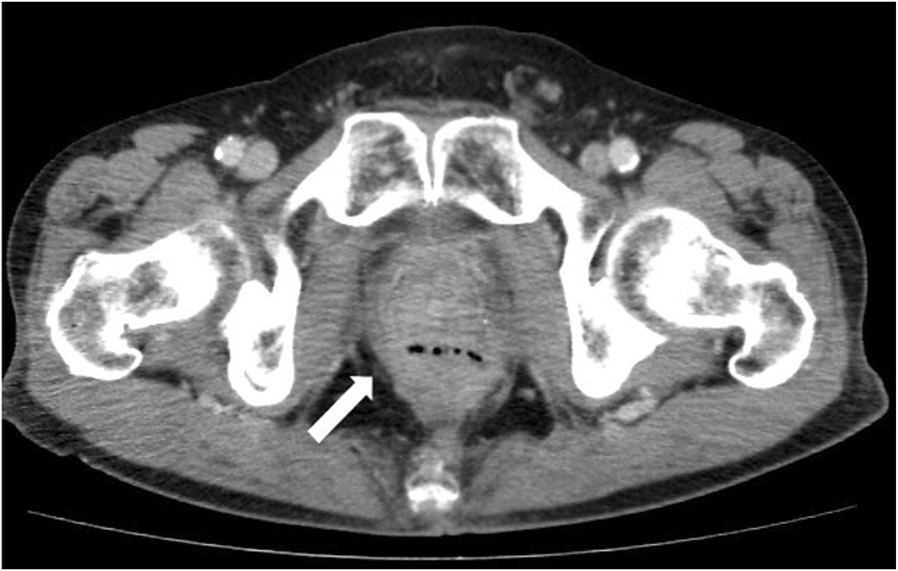
Computed tomography scan of the abdomen revealed a small amount of extraluminal air between the prostate and rectum, adjacent to the anastomotic site (white arrow)

**Fig. 2:**
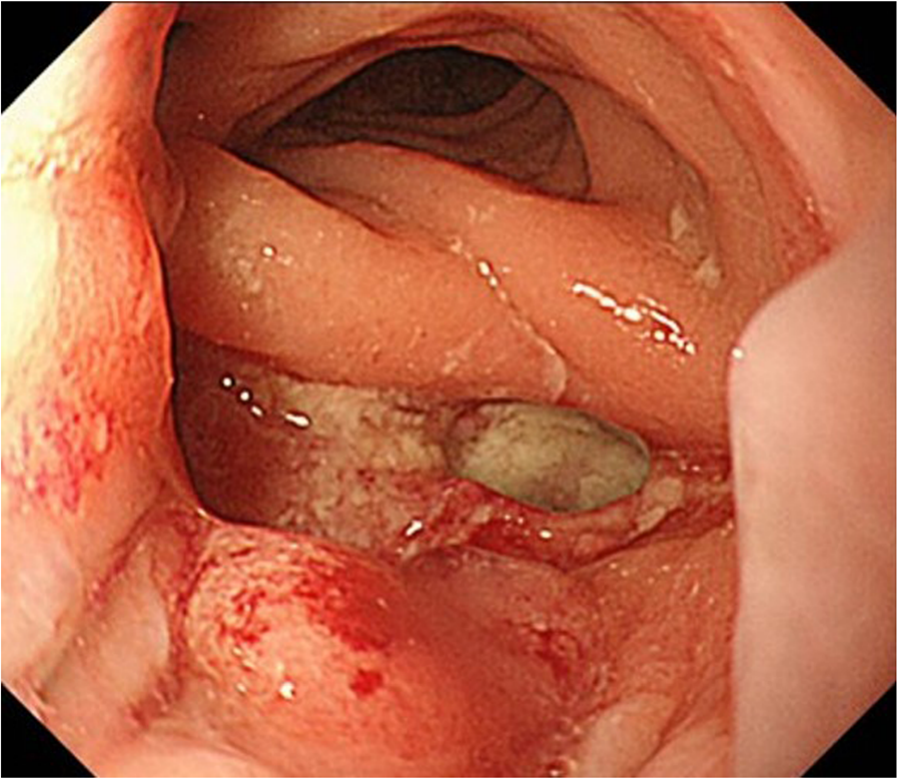
Ulceration and fistula formation are observed at the site of the colonic anastomosis on colonoscopy

**Fig. 3:**
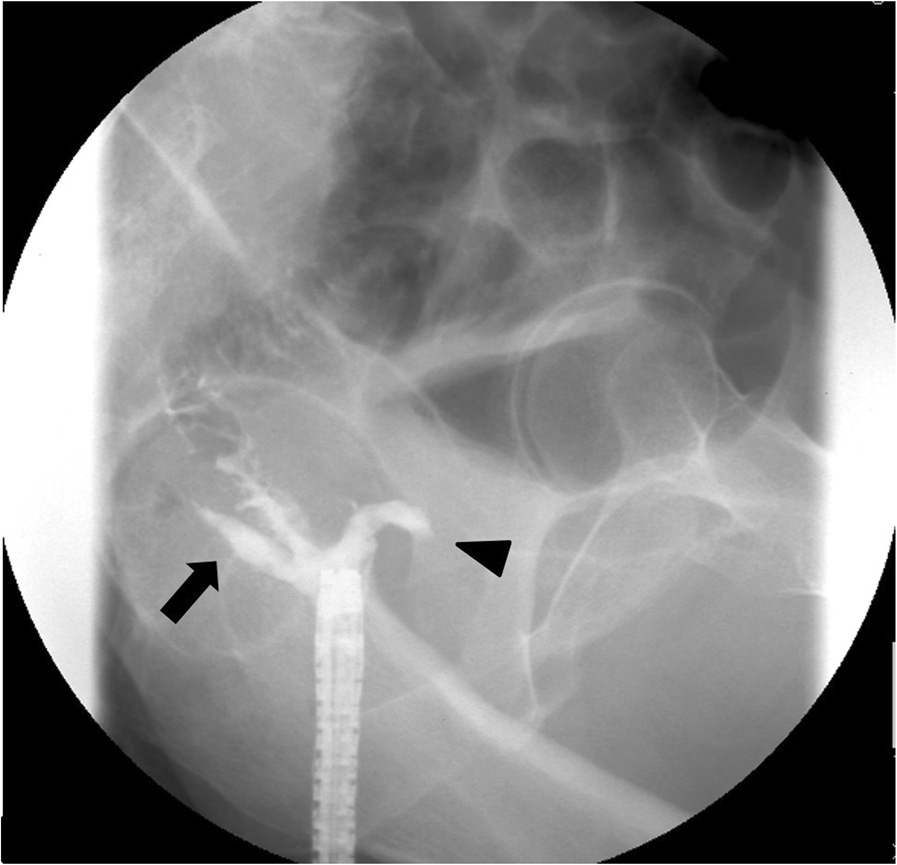
Fistula formation was observed at the anastomotic site after low anterior resection on contrast radiography. Arrow head and arrow indicate fistulae which go to the anterior and posterior side of the anastomosis, respectively. There was no anastomotic-urethral fistula seen on contrast enema

During follow-up, multiple lymph node metastases in the para-aortic and supraclavicular regions were found 20 months after resection and chemotherapy was given, including 14 days of oral capecitabine, 1 day of oxizaliplatin (CAPOX), and bevacizumab. Bevacizumab (7.5 mg/kg) was administered intravenously on day 1 for 1 cycle. CAPOX+bevacizumab was continued for 3 years for a total of 33 cycles of CAPOX (combined with 23 cycles of bevacizumab). Progression of lymph node metastases was noted and the chemotherapy regimen was changed. Second-line chemotherapy included 14 days of oral capecitabine, 1 day of irinotecan (XELIRI), and bevacizumab. Three cycles of XELIRI+bevacizumab (7.5 mg/kg) were continued over 3 months. One month after the last course of chemotherapy (5 years after resection), the patient presented with pneumaturia and fecaluria and described three previous episodes of hematochezia. The patient had no other symptoms associated with altered bowel habits. Laboratory tests demonstrated signs of mild inflammation (white blood cell count 11,200 μL, C-reactive protein 2.2 mg/dL). A small amount of extraluminal air between the prostate and rectum, adjacent to the anastomotic site, was observed on computed tomography (CT) scan (Fig. 1), which was not noted on the previous CT scans. Routine surveillance colonoscopy was not performed after the initial surgery as care was focused on management of multiple lymph node metastases. The latest colonoscopy revealed ulceration and fistula formation at the anastomotic site (Fig. 2). Contrast radiography was consistent with a fistula at the anastomotic site (Fig. 3). Only granulation tissue, including inflammatory changes, was seen in biopsies taken from the anastomotic site. Colonoscopy, contrast radiography, and cystoscopy demonstrated no recurrent tumor or obvious anastomotic-urethral fistula at the anastomosis, but an anastomotic-urethral fistula was suspected based on symptoms including pneumaturia and fecaluria. Transverse colostomy was performed and has not been reversed because of the continued need for chemotherapy. At this time, the patient has no symptoms associated with a leak and CT scan shows no air around the anastomotic site.

## Discussion

The present patient developed a delayed anastomotic leak associated with bevacizumab treatment for metastatic rectal cancer, which presented more than 60 months after a very low anterior resection with primary anastomosis. Although multiple para-aortic lymph node metastases were observed, there was no recurrence of the tumor at the anastomotic site when the leak occurred. Previous treatment with preoperative CRT may be associated with this delayed anastomotic leak. This patient highlights a rare late adverse event at the anastomotic site associated with bevacizumab treatment and preoperative CRT.

Anastomotic leak is one of the most serious complications following colorectal surgery, and generally occurs within 30 days postoperatively. However, recent studies have reported that anastomotic leaks can also occur more than 30 days after surgery. Risk factors for early anastomotic leak following rectal surgery include a low site of anastomosis, male gender, and the presence of intraoperative difficulties, which may correlate with the degree of surgical difficulty [12–14]. Delayed anastomotic leak does not seem to be associated with technical factors. The risk factors for delayed anastomotic leak include female gender, low anastomosis, preoperative CRT, and fistula formation [15, 16]. Of these risk factors for the development of delayed anastomotic leak, the present patient had a low anastomosis and preoperative CRT. Radiotherapy affects wound healing by inducing vascular injury, inhibiting angiogenesis and impairing fibroblasts [17]. Anastomosis creates a region of marginal vascularization, which may incur further ischemic damage by radiotherapy. Delayed anastomotic leak may occur regardless of bevacizumab administration.

Gastrointestinal perforation is a well-described complication of bevacizumab administration that has been reported in up to 2% of patients with CRC and can occur at any time during treatment [18]. The anastomosis is a well-known site for perforation. An intact primary tumor, concomitant use of non-steroidal anti-inflammatory drug, advanced age, and previous radiotherapy were found to be significant risk factors for the development of gastrointestinal perforation associated with bevacizumab treatment [19, 20]. Bevacizumab-related delayed anastomotic leak is extremely rare.

There have only been nine patients treated with bevacizumab in six reports of delayed anastomotic leak occurring more than 12 months postoperatively [6–11]. The clinical features of all 10 patients, including the present patient, are summarized in Table 1. All patients had rectal cancers and underwent preoperative CRT. The leaks occurred after beginning bevacizumab treatment, and 40% had a previous history of anastomotic leak. The median time to detecting the leak after the initial surgery was 30 months in the previously reported nine patients. We compared the duration of bevacizumab treatment between patients with a delayed leak occurring more or less than 30 months after primary anastomosis. The median duration of bevacizumab treatment in six patients with a greater than 30 month interval was longer (20 weeks) than the median in four patients with a less than 30 month interval (6 weeks). The interval between the last bevacizumab dose and leak was not different in the two groups. In the present patient, the leak occurred after 60 months, the longest reported interval following resection, and 3 years, the longest duration of bevacizumab treatment of the 10 patients reviewed. Long-term follow-up at the anastomotic site is needed for patients who receive bevacizumab treatment. Significant risk factors associated with the development of delayed anastomotic leak include a low anastomotic site, preoperative CRT, and bevacizumab treatment.

**Table 1:** Clinical features of ten patients with delayed anastomotic leak associated with bevacizumab treatment

Report	Age/gender^1^	Primary tumor	Preoperative radiation therapy	Previous history of anastomotic leak	Anastomotic complication	Interval between initial surgery and leak (months)	Duration of bevacizumab treatment	Interval between last bevacizumab dose and leak
Adenis et al. [9]	50/F	Rectum	Yes	Yes	Leak ileovaginal fistula	22	1 month (2 doses)	< 1 month
Ley et al. [7]	72/F	Rectum	Yes	No	Leak rectovaginal fistula	30	6 months	< 1 month
August et al. [6]	58/M	Rectum	Yes	Yes	Leak	26	6 weeks (3 doses)	Not described
August et al. [6]	74/F	Rectum	Yes	No	Leak	33	20 weeks	4 months
Bege et al. [8]	46/M	Rectum	Yes	Yes	Leak	52	Not described	1 week
Bege et al. [8]	54/M	Rectum	Yes	Yes	Leak	57	Not described	2 weeks
Bege et al. [8]	51/M	Rectum	Yes	No	Leak	21	Not described	2 weeks
Borzomati et al. [10]	68/M	Rectum	Yes	Yes	Leak	33	24 weeks	2 weeks
O’Hare et al. [11]	56/F	Rectum	Yes	No	Leak	17	6 weeks (3 doses)	1 month
Present patient	78/M	Rectum	Yes	No	Leak	60	3 years (26 doses)	1 month

^1^*F* female, *M* male

In the present patient, the delayed anastomotic leak was detected 3 cycles after beginning an irinotecan-based regimen. Irinotecan is also suspected to be associated with the development of delayed anastomotic leaks, but usually causes a range of toxicities including diarrhea and neutropenia, and the incidence of gastrointestinal perforation is much less than with bevacizumab-based treatment [5, 21, 22]. In addition, no previous report of delayed anastomotic leak due to irinotecan was identified. After creating the transverse colostomy for treatment of the leak, irinotecan plus anti EGF receptor drug has been given. There is no evidence of further leak. In consideration of previous reports, bevacizumab is the most likely drug to be associated with development of the delayed anastomotic leak in the present patient.

**Fig. 4:**
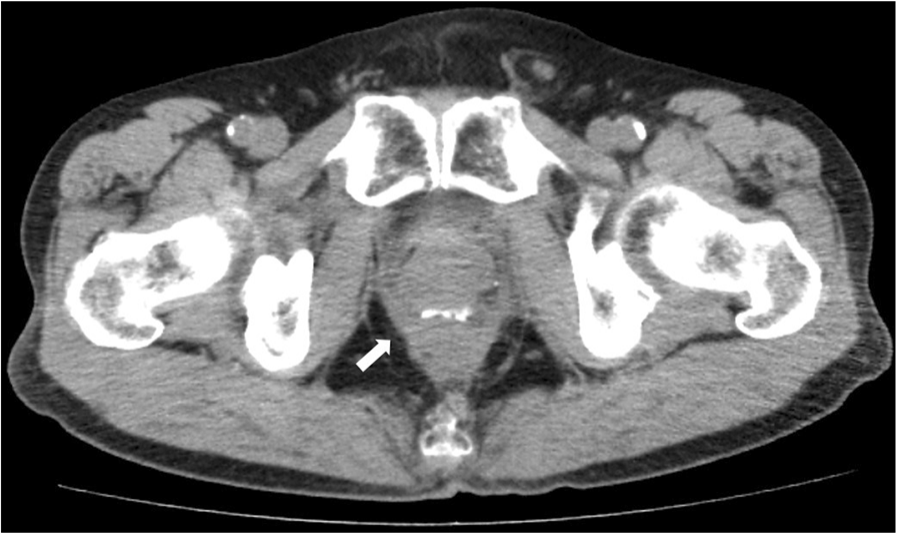
Computed tomography scan 3 months before the patient presented with symptoms of a leak shows a circumferential staple line at the anastomotic site

Previous CT scan showed that the circumferential staple line was observed at the anastomotic site (Fig. 4) but was no longer apparent at the time the leak was noted (Fig. 1). Staples might be spontaneously extruded at the anastomotic site during bevacizumab treatment. Some studies demonstrated possible deleterious effects of agents targeting VEGF on healing of colonic anastomoses [23, 24]. Stapled anastomoses are commonly used in rectal cancer surgery and granulation tissue develops around the anastomotic site. There may be ongoing healing and tissue remodeling for years following a low anterior resection in a patient who had received radiation therapy [17]. Bevacizumab may interfere with the ongoing healing process, and an inflamed wound is more at risk of complications from VEGF inhibition at any time during such treatment.

## Conclusions

A delayed anastomotic leak with fistula formation after low anterior resection for rectal cancer associated with bevacizumab treatment and preoperative CRT must be considered. Signs or symptoms suggestive of anastomotic complications should be evaluated during bevacizumab treatment, even long after the initial surgery.

## Abbreviations

5-FU: 5-Fluorouracil
CRC: Colorectal cancer
CRT: Chemo-radiotherapy
CT: Computed tomography
Gy: Gray
VEGF: Vascular endothelial growth factor
